# Do Eicosapentaenoic Acid and Docosahexaenoic Acid Have the Potential to Compete against Each Other?

**DOI:** 10.3390/nu12123718

**Published:** 2020-12-02

**Authors:** Anandita Pal, Adam H. Metherel, Lauren Fiabane, Nicole Buddenbaum, Richard P. Bazinet, Saame Raza Shaikh

**Affiliations:** 1 Department of Nutrition, Gillings School of Global Public Health and School of Medicine, The University of North Carolina at Chapel Hill, 170 Rosenau Hall, CB# 7400, 135 Dauer Drive, Chapel Hill, NC 27516, USA; dita991@email.unc.edu (A.P.); fiabane.lauren@gmail.com (L.F.); nicole_buddenbaum@med.unc.edu (N.B.); 2 Department of Nutritional Sciences, Medical Sciences Building, 5th Floor, Room 5358, University of Toronto, 1 King’s College Circle, Toronto, ON M5S 1A8, Canada; adam.metherel@utoronto.ca (A.H.M.); richard.bazinet@utoronto.ca (R.P.B.)

**Keywords:** omega-3 fatty acids, EPA, DHA, arachidonic acid, membrane, western diet

## Abstract

Eicosapentaenoic acid (EPA) and docosahexaenoic acid (DHA) are n-3 polyunsaturated fatty acids (PUFAs) consumed in low abundance in the Western diet. Increased consumption of n-3 PUFAs may have beneficial effects for a wide range of physiological outcomes including chronic inflammation. However, considerable mechanistic gaps in knowledge exist about EPA versus DHA, which are often studied as a mixture. We suggest the novel hypothesis that EPA and DHA may compete against each other through overlapping mechanisms. First, EPA and DHA may compete for residency in membrane phospholipids and thereby differentially displace n-6 PUFAs, which are highly prevalent in the Western diet. This would influence biosynthesis of downstream metabolites of inflammation initiation and resolution. Second, EPA and DHA exert different effects on plasma membrane biophysical structure, creating an additional layer of competition between the fatty acids in controlling signaling. Third, DHA regulates membrane EPA levels by lowering its rate of conversion to EPA’s elongation product n-3 docosapentaenoic acid. Collectively, we propose the critical need to investigate molecular competition between EPA and DHA in health and disease, which would ultimately impact dietary recommendations and precision nutrition trials.

## 1. Introduction

The long chain n-3 polyunsaturated fatty acids (PUFAs) eicosapentaenoic (EPA, 20:5) and docosahexaenoic acid (DHA, 22:6) are dietary nutrients that are consumed in low abundance in the Western diet [1]. N-3 PUFAs, obtained from marine oils and supplements, have attracted considerable attention given their potential to improve various health outcomes. N-3 PUFAs are clinically approved for treating hypertriglyceridemia associated with metabolic syndrome, obesity, type 2 diabetes, and cardiovascular disorders. Recently, a highly pure EPA ethyl ester (Vascepa^®^) was approved by the Food and Drug Administration for lowering the risk of cardiovascular disease in select clinical populations [2]. This overturned conclusions from some randomized clinical trials and meta-analyses that n-3 PUFAs (usually administered as mixtures) have no therapeutic benefits for cardiovascular pathologies [3]. The effects with pure EPA are notable since a recent randomized clinical trial (STRENGTH) using mixed EPA/DHA carboxylic acids (EPANOVA) was suspended as cardiovascular benefits were not detected [4]. This raises the intriguing possibility of DHA negating the effects of EPA, although the suspension of the EPANOVA study is likely driven by many factors such as product form, the placebo, issues with compliance, and demographics [4].

In contrast to the aforementioned examples on cardiometabolic risk, the potential to use n-3 PUFAs for other physiological outcomes remains to be established. In particular, the utility of n-3 PUFAs for controlling chronic inflammation driven by environmental and nutritional stressors or treating conditions such as depression is unclear, which could be due to differences in the bioactivity between EPA and DHA. To exemplify, a recent meta-analysis of placebo controlled randomized clinical trials demonstrated that n-3 PUFAs had some benefits for depressive symptoms, which were more pronounced for a higher concentration of EPA, but not DHA [5]. Sublette et al. suggested that the amount of EPA present in excess of DHA leads to a therapeutic outcome and that the effects of EPA reached statistical significance in their study only when administered in levels that exceeded DHA supplementation [6]. To further exemplify, a very recent study suggested that EPA impairs learning and memory in mouse models, which could be reversed with DHA at specific ratios relative to EPA [7]. Thus, there is a need to investigate the effects that EPA and DHA have on one another for differing functional endpoints.

There is strong evidence for complementary effects of EPA and DHA for differing physiological endpoints [8,9]. Herein, we propose the novel concept that EPA and DHA in some cases could also oppose each other, which warrants further investigation at a molecular level. We first review evidence to suggest that membrane EPA and DHA could compete for incorporation into the membrane phospholipid pool and thereby differentially impact the abundance of n-6 PUFAs that are highly prevalent in the Western diet. This would strongly impact downstream signaling and turnover to metabolites of inflammation initiation and resolution in select cells and tissues. We then cover differences between EPA and DHA on membrane biophysical properties including formation of lipid rafts, which regulate intracellular signaling and thereby gene expression. Finally, we discuss recent evidence on DHA regulating the levels of membrane EPA and its elongation product n-3 docosapentaenoic acid (DPAn-3, 22:5), which is also a precursor for metabolites that resolve inflammation [10].

## 2. Could EPA and DHA Compete to Incorporate into the Membrane Phospholipidome?

Dietary intake of EPA/DHA-enriched marine oils increases the levels of EPA and DHA across tissues in a dose- and time-dependent manner [11]. The increase in EPA and DHA upon dietary supplementation results in a significant replacement of differing fatty acids with a prominent impact on the levels of n-6 PUFAs. As an example, incorporation of EPA/DHA after one year of supplementation with increasing doses of oily fish resulted in displacement of oleic acid and the n-6 PUFAs linoleic acid, di-homo-γ-linolenic acid, and arachidonic acid in plasma phosphatidylcholines (PC). In the plasma cholesterol ester pool, increasing levels of EPA/DHA lowered linoleic acid and γ-linolenic acid, whereas EPA/DHA lowered oleic acid levels in triacylglycerols [12]. However, it remains unclear if EPA and DHA differentially impact the displacement of n-6 PUFAs, particularly upon varying the ratio of EPA to DHA. Specifically, would the ability of EPA and DHA to differentially replace n-6 PUFAs be driven by any potential competition between the two long chain n-3 PUFAs for esterification into membrane phospholipids?

There is evidence that EPA and DHA have differing effects on the specific fatty acids that are replaced. We summarize a few select studies. One study reported that EPA (1.8 g per day), but not DHA (1.8 g per day), lowered plasma oleic acid levels after 6 weeks of intervention [13]. Two studies published using the same cohort of individuals distinguished the effects of DHA and EPA supplementation on arachidonic acid levels [14,15]. Specifically, following twelve weeks of 3 g per day of EPA supplementation, arachidonic acid levels decreased in erythrocytes [15] but not total plasma [14], while an identical DHA supplementation protocol lowered arachidonic acid in both pools. Another study demonstrated that 3.8 g of EPA per day for seven weeks lowered arachidonic acid in plasma phospholipids [16]. These studies suggest competition between EPA and arachidonic acid for the PC pool, and possibly the phosphatidylethanolamine (PE) pool, but not the larger triacylglycerol pool. Some have attributed the arachidonic acid lowering effect of n-3 PUFAs to a reduction in arachidonic acid synthesis; however, to our knowledge this has never been demonstrated directly. In fact, DHA feeding (2% of total fatty acids) in rats for up to eight weeks resulted in no statistical change with in vivo arachidonic acid synthesis-secretion rates when compared with controls [17]. Furthermore, DHA feeding appears to regulate brain n-6 docosapentaenoic acid levels while leaving arachidonic acid relatively unchanged [18,19]. However, the effect of DHA feeding on arachidonic acid levels appears to be tissue specific [18].

Along with the effects on arachidonic acid and oleic acid as described above, there is also evidence demonstrating that EPA and DHA differentially lower the levels of linoleic acid (18:2, n-6). For instance, in subjects with obesity and subclinical inflammation, EPA lowered the levels of linoleic acid to a greater extent than DHA, despite the fact that DHA increased the omega-3 index to a greater extent than EPA [20]. Similarly, another study reported that EPA lowered linoleic and arachidonic acid levels in plasma to a much greater extent (about 2-fold) than DHA. In this study, it was also observed that EPA lowered platelet phospholipid DHA levels by 28% [21]. In a mouse model of obesity, it was reported that EPA, but not DHA, lowered total B lymphocyte levels of linoleic acid [22]. On the contrary, administration of EPA or DHA to a lymphoma cancer line had no effect on linoleic acid levels [23,24]. Using well defined culture and animal models, future studies need to elucidate how modifications in the ratio of EPA to DHA can lower n-6 PUFAs and if this is driven by competition between EPA and DHA for the membrane phospholipid pool.

An additional layer of complexity is that EPA and DHA are unlikely to end up in the same type of phospholipid within a particular cell type. Thus, it is conceivable that EPA and DHA could influence uptake of each other upon esterification into differing phospholipids. Several labs report that EPA and DHA are differentially incorporated into distinct membrane phospholipids [25]. The uptake of EPA and DHA is selective and varies between PC, PE, phosphatidylserines (PS), and phosphatidylinositols (PI) [26,27]. EPA has been reported to be preferentially incorporated into PC followed by PE as compared to DHA, which has been found to be highest in PE followed by PC in erythrocyte membranes of healthy humans [28]. Notably, in platelets, EPA has also been found to inhibit the incorporation of DHA and has increased its relative acylation into PE at the expense of PC [29]. Since it is well established that PC is more abundant in the outer leaflet of the plasma membrane and PE and PS is more enriched in the inner membrane, EPA could be preferentially located in the outer membrane and DHA in the inner membrane, which would have strong implications for plasma membrane structure and signaling.

A fish oil supplementation and washout study found that EPA increased and decreased in erythrocyte membranes more rapidly compared to DHA [30]. This slower DHA incorporation could be the result of an ATP-dependent trans-bilayer process required to move DHA to the high PE-containing inner leaflet [31]. Nevertheless, increases in EPA specific to a particular phospholipid species or a location within that phospholipid species (i.e., *sn*-1 or *sn*-2) may result in differing availabilities for release and entry into the eicosanoid pool and other pathways within tissues, which may then affect the biological response to n-3 PUFA supplementation. However, due to the limited availability of such studies, substantially more research must be conducted to investigate this nascent hypothesis.

Differences in substrate affinities for several enzymes could drive the differential incorporation of EPA and DHA into the phospholipid pool. For instance, DHA accumulation in phospholipids may take precedence over EPA in the Kennedy pathway. This has been corroborated by the known preference of the following two enzymes towards DHA [32,33]: (1) cytidine diphosphate-ethanolamine:diacylglycerol ethanolaminephosphotransferase, which converts DAG to PE, and (2) PE *N*-methyltransferase, which converts PE to PC. Acyl CoA: diacylglycerol acyltransferase (DGAT) is a microsomal enzyme that plays a central role in the metabolism of cellular glycerolipids [34]. DGAT catalyzes triacylglycerol synthesis by using DAG and fatty acyl CoAs as its substrates. EPA and EPA-CoA, being poor substrates for DGAT, reduce triacylglycerol synthesis [35,36]. Additionally, both PS synthase and PS-decarboxylase enzymes, which synthesizes PS from either PC or PE and decarboxyates PS to yield PE, respectively, exhibit substrate preference for DHA-containing phospholipids, which may explain the enrichment of DHA in both the PE and PS pools within the brain [37]. What remains unclear is whether the presence of EPA could hinder DHA accumulation in PS and PE pools, and is an area for future investigation.

Another area for future research is whether potential competition between EPA and DHA for the membrane phospholipid pool will control downstream biosynthesis of specialized pro-resolving mediators (SPMs), which are potent autacoids that have a critical role in inflammation resolution, infectious outcomes, and tissue regeneration [38,39]. EPA and DHA undergo processing via several cytochrome P450, lipoxygenase (LOX)-, and cyclooxygenase (COX)-mediated pathways to metabolize into biologically active signaling molecules. Liquid chromatography–tandem mass spectrometry identified several unique eicosapentaenoic acid derived E-series resolvins and docosahexaenoic acid derived D-series resolvins, protectins, and maresins, which stimulate specific 7-transmembrane G-protein coupled receptors at pico- to nanogram levels to control a variety of cellular functions [40,41]. Notably, it is critical to determine whether biosynthesis of EPA- or DHA- derived metabolites impact arachidonic acid and linoleic acid-derived metabolite levels. Arachidonic acid-derived eicosanoids such as prostaglandin E2 (PGE_2_) drive subsequent pro-resolution responses [42,43]. There is also biochemical evidence that differing fatty acids including n-3 PUFAs are competing with arachidonic acid for binding to COXs [44]. Furthermore, there is a need to study the temporal component between EPA and DHA on downstream metabolite biosynthesis.

## 3. Would Modifying the EPA to DHA Ratio Impact Membrane Biophysical Organization?

N-3 PUFA acyl chains control bilayer properties that are important for signaling. These properties include lateral pressure, microviscosity, curvature, permeability, elasticity, microdomain formation, hydrophobic match, membrane fluidity, lateral pressure, compressibility, and phospholipid flip-flop [45,46,47,48,49,50]. The multiple double bonds in PUFA acyl chains are responsible for the modification to bilayer organization [47]. Unsaturated fatty acids exhibit a more curved structure as compared to saturated fatty acids due to the presence of cis double bonds [51]. Relative to the straight chain structure of saturated fatty acids, which allow them to be packed easily into the membrane bilayer, n-3 PUFA acyl chains generally decrease microviscosity and packing [51,52,53,54]. Measurements with a wide variety of biophysical techniques such as solid state ^2^H NMR demonstrate that PUFA acyl chains have an important role in lowering the membrane hydrocarbon order [55,56,57].

The difference in hydrocarbon length and number of double bonds between EPA and DHA will differentially control membrane bilayer properties [58]. EPA notably occupies a distinct location in the membrane as it intercalates with its long axis parallel to the phospholipid acyl chains [58]. EPA’s hydrocarbon length and double bonds enhance intercalation into the core of the membrane bilayer, which may have physiological implications for humans [59,60]. EPA’s structure allows it to be inserted into cellular membranes in an extended conformation that contributes to enhanced scavenging of reactive oxygen species [50]. X-ray diffraction studies demonstrate that membrane hydrocarbon core electron density increases with EPA as compared with DHA [50]. Thus, the structural differences between EPA and DHA are likely to differentially impact functional outcomes such as scavenging of reactive oxygen species.

A major target of n-3 PUFAs are lipid domains known as lipid rafts [61,62,63]. Lipid rafts are 10–200 nm liquid-ordered membrane domains characterized by tightly packed cholesterol and sphingolipids that serve as transient platforms for efficient cellular signaling [64]. EPA and DHA partition differently into raft and non-raft domains [65]. Some studies suggest that DHA, compared to EPA, has a greater tendency to accumulate within lipid rafts. DHA’s high acyl chain flexibility and rapid conformational changes have been proposed to interfere with the packing of lipid raft associated cholesterol, which is a highly rigid structure [66]. DHA, unlike EPA, appears to modify the size of cholesterol-rich domains in biomimetic membranes and cells [66,67]. To exemplify, a heteroacid DHA-containing PC was incorporated directly into sphingolipid/cholesterol-enriched domains modeling lipid rafts; in contrast, the EPA-containing heteroacid PC was largely incorporated into non-rafts [65]. Follow-up molecular dynamic simulations demonstrated that the EPA-containing PC was far more disordered in structure compared to the DHA-containing PC [68]. In studies with lymphomas and primary B lymphocytes, EPA and DHA exerted differential effects on cholera-toxin induced lipid raft GM1 clustering [23]. DHA completely disrupted lipid raft clustering whereas EPA had no effect. This was also observed in vivo where B cells isolated from mice consuming a DHA but not EPA enriched diet diminished lipid raft clustering [69].

We hypothesize that increasing EPA levels could prevent the uptake and esterification of DHA into plasma membrane phospholipids and thereby prevent the raft-disrupting effects of DHA. Our hypothesis does not preclude the notion that EPA remains a membrane fluidizer that is largely localized to non-raft domains. Thus, administering both fatty acids together in pre-clinical and clinical studies could confound outcomes assuming that the effects on the plasma membrane have functional consequences. Additionally, it remains unclear if excess EPA could diminish the effects of DHA on plasma membrane raft-mediated signaling and gene expression. Potential opposing effects of EPA and DHA on membrane molecular order and raft formation are also likely influenced by metabolic demands of the cell [70]. To exemplify, a recent study showed that DHA remodeled plasma membrane composition in vitro and in vivo to lower plasma membrane packing, which was compensated by an increase in the concentration of cholesterol and saturated fatty acids [70].

**Figure 1:**
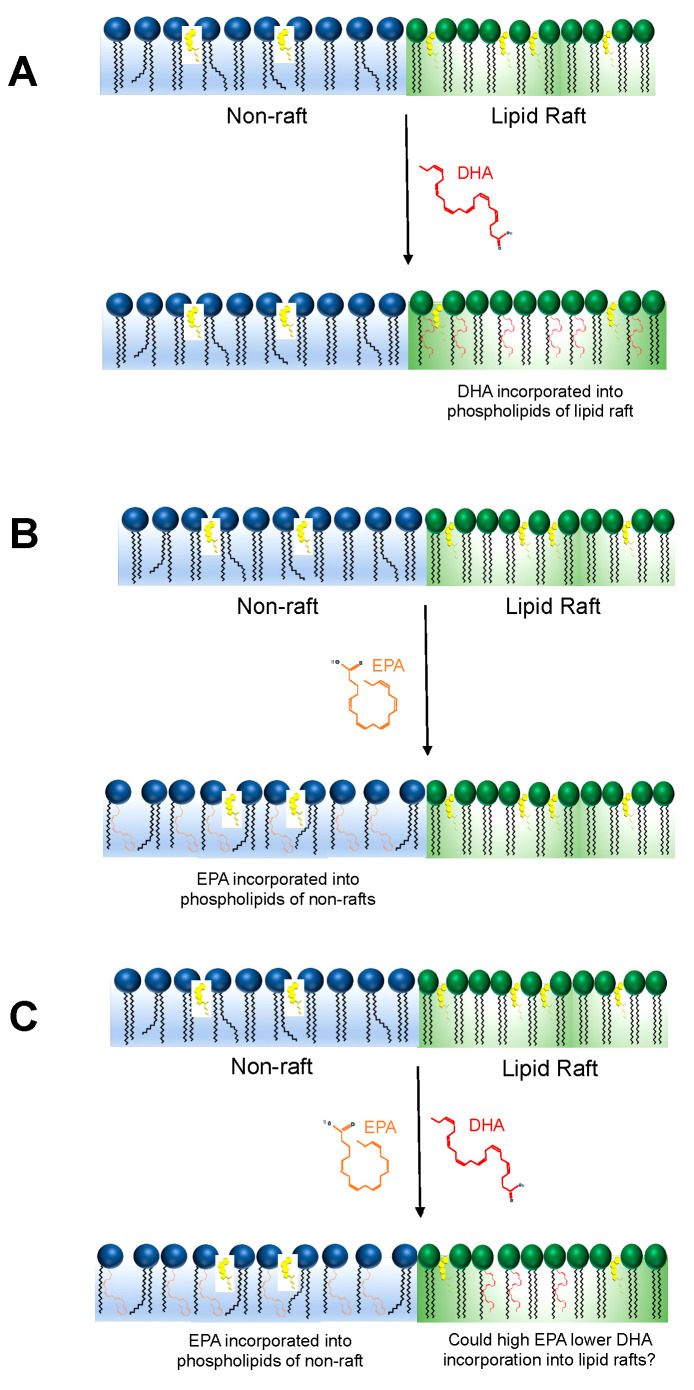
EPA (eicosapentaenoic acid) and DHA (docosahexaenoic acid) exert differential effects on lipid rafts in some cell types. (**A**) DHA can directly infiltrate lipid rafts to modify their biophysical organization whereas (**B**) EPA largely avoids interactions within lipid rafts and prefers the disordered non-raft region. (**C**) DHA incorporation and disruption of lipid raft size and stability may be influenced by the availability of EPA in the membrane, which is an area for future investigation.

In Figure 1, we summarize the effects of EPA and DHA on lipid rafts. DHA incorporates directly into lipid rafts (Figure 1A), whereas EPA avoids lipid rafts (Figure 1B). However, whether competition between EPA and DHA for occupancy into membrane phospholipids controls formation and function of lipid rafts remains unknown. Would high EPA interfere with the ability of DHA to incorporate and target lipid raft biophysical organization? (Figure 1C). This is a major gap in knowledge that warrants further investigation by integrating biophysical studies using biomimetic membranes with in vivo models. Furthermore, it is critical to establish the signaling effects of modifying the ratio of EPA to DHA upon targeting of lipid rafts.

## 4. Does Dietary DHA Inhibit EPA Metabolism at the Level of the Membrane?

DHA supplementation alone consistently results in 49–130% higher EPA levels across a variety of species, blood fractions, and tissues [71], a phenomenon that has persistently been attributed to the direct conversion of DHA to EPA (known as retroconversion) via a peroxisomal β-oxidation [72]. However, recent evidence utilizing compound-specific isotope analysis to determine the source of the increase in EPA following DHA feeding has clearly identified alpha-linolenic acid—not DHA—as the dietary/metabolic precursor of the increase in EPA in both rodents [73] and humans [14]. These findings identify a possible feedback inhibition signal by DHA to reduce EPA metabolism and may explain the accumulation of EPA in the membrane upon EPA/DHA supplementation. However, further studies are essential to identify the mechanism of this inhibition and the effect on downstream products of EPA. One product of EPA metabolism that is clearly lowered following DHA supplementation is DPAn-3 [74,75], which suggests that DHA may act to inhibit the activity of elongase 2 and/or elongase 5 for the 2-carbon elongation process converting EPA to DPAn-3. However, a reduction in DPAn-3 levels could be explained by a reduction in DPAn-3 synthesis, an increase in metabolic consumption of DPAn-3, or a combination of both.

There has been some emphasis on the beneficial effects of high-EPA or EPA-only supplements in comparison to high-DHA or DHA-only supplements, which has been highlighted by meta-analyses, particularly as it pertains to depression [5,76,77]. This understanding combined with the recent knowledge that DHA appears to inhibit EPA metabolism offers some potentially interesting mechanistic explanations for the potential dichotomy between the health benefits of the two long chain n-3 PUFAs [71]. While DHA supplementation alone appears to be limited to an approximate doubling in EPA levels, EPA supplementation can result in multiple fold increases in EPA levels. This disproportionate effect suggests alternative mechanisms to explain DHA’s effect on EPA levels. Notably, the dietary and blood/tissue DHA levels that begin to exert this effect, how and where EPA accumulates, and the specific metabolic pathways involved remain to be investigated.

Numerous pathways could be affected by the previously described DHA-initiated inhibition of EPA metabolism, including those that produce *n*-acylethanolamines and eicosanoids. The eicosanoid pathway offers an appealing explanation for the beneficial effects of high EPA supplements compared to high DHA supplements. This pathway begins with the release of a 20-carbon fatty acid from membrane phospholipids, primarily arachidonic acid or EPA by phospholipase A_2_, which is then oxygenated to eventually form prostaglandins or thromboxanes by COX-2 and leukotrienes by LOX, among others [78]. Human primary monocyte-derived macrophages and RAW264.7 macrophage cell lines treated acutely with DHA resulted in a concentration-dependent increase in phospholipase-mediated arachidonic acid release from phospholipids, resulting in activation of COX-2 and up to 1000-fold increases in PGE_2_ [79]. This potentially large increase in the production of the more inflammatory n-6-derived eicosanoids may offset the perceived beneficial effect of increased docosanoid production from DHA and n-3-derived eicosanoids from EPA. However, this is an acute response in isolated cells that may not be indicative of the lipid mediator response to a more chronic dietary exposure to EPA or DHA.

Rats fed a high DHA or a high EPA diet for up to 6 weeks both demonstrated significantly lower PGE_2_ levels in spleen leukocytes compared to control [80]. Similarly, mice fed either 1% EPA or 1% DHA in total fat diets for 2 weeks showed no changes in total brain arachidonic acid-derived epoxides, hydroxides, prostaglandins, or thromboxanes, and only a small decrease in arachidonic-derived epoxides of the heart as compared to the control [81]. In the same study, EPA feeding compared to DHA feeding resulted in significantly higher levels of EPA-derived epoxides and hydroxides in both heart and brain, with levels only marginally higher or identical in DHA fed animals compared to controls [81]. Fish oil supplementation in which EPA levels were higher than DHA has shown no significant changes in arachidonic acid-derived prostaglandins, leukotrienes, and others, but significant increases in EPA-derived eicosanoids and DHA-derived docosanoids [82,83]. Therefore, despite possible increases in arachidonic acid release from the phospholipid membrane in response to acute DHA exposure, large increases in EPA following more chronic dietary EPA/DHA may provide enough competition with arachidonic acid for the COX and LOX enzymes to result in a lowered inflammatory profile. This shift towards n-3-derived eicosanoids with EPA feeding compared to DHA feeding may help to explain some of the benefits of high EPA compared to DHA supplements. Under such a situation, we can reasonably hypothesize that DHA-predominant n-3 PUFA supplements do not alter arachidonic acid derived eicosanoids while EPA-predominant supplements increase EPA-derived eicosanoids.

Overall, it remains unclear if there is potential for competition upon DHA intake on EPA metabolism, but is an area of investigation that is just emerging. Establishing the molecular membrane-based underpinnings of EPA and DHA at a metabolic level will provide critical information to guide future randomized clinical trials with the two fatty acids.

## 5. Future Directions

There is a critical need to distinguish the membrane-based molecular effects of DHA in the presence of increasing EPA and vice versa. Few studies investigate EPA- and DHA-enriched oils separately in the context of membrane biology, but more in-depth pre-clinical molecular studies using biochemical and biophysical approaches could set the basis for specific ratios of EPA to DHA to be investigated in clinical studies for specific health outcomes. Ultimately, this basic molecular knowledge will drive precision nutrition studies in which EPA and DHA are investigated at defined ratios that are optimal for human health and disease. We acknowledge that challenges will arise including accounting for background levels of EPA vs. DHA in circulation (and differing tissues) and the design of supplements with defined ratios of EPA and DHA. Furthermore, host genetics are likely to determine the metabolism of EPA and DHA along with their downstream metabolites [84,85]. In addition, it remains unknown if the potential competition between EPA and DHA would arise under conditions in which n-3 PUFA intake is low (as in the typical Western diet) and under conditions in which n-3 PUFA intake is high.

Rodent models in future studies will provide insight on how modifying the EPA/DHA ratio may impact the concentration of EPA/DHA in the membrane and thereby downstream metabolites of inflammation initiation and resolution. Future experiments will be able to assess if the proposed hypothesis is tissue-, cell-, and organelle-specific. For instance, studies are emerging to potentially suggest a competitive effect of EPA/DHA on depression or memory/learning as described above. However, an in depth analysis is critical to elucidate how EPA/DHA competes in the membrane of specific cells in defined tissues to control signaling events or downstream metabolite biosynthesis. This systematic approach could then guide translational experiments in the future.

## 6. Conclusions

EPA may counter-regulate the physiological effects of DHA (and vice versa) in select tissues by competing for occupancy in the phospholipid membrane pool to differentially lower n-6 PUFAs, modulate membrane biophysical properties such as the formation of lipid rafts, and hinder metabolism through feedback inhibition. As described above, this notion is underscored by some recent studies that suggest that EPA and DHA may counter-regulate each other. Further investigation at a molecular level on these three mechanisms is critical to ultimately make better dietary recommendations and design precision clinical trials to mitigate inflammation-mediated chronic diseases associated with the Western diet.
